# A Conversation with Tzahi Cath

**DOI:** 10.1021/acscentsci.4c02146

**Published:** 2024-12-30

**Authors:** XiaoZhi Lim

With population growth and climate change both stressing freshwater supplies, demand for the precious resource could exceed supply by some 40% by 2030, according to a 2016 United Nations estimate. Growing up on a farm in Israel, Tzahi Cath became attuned to the problem of freshwater scarcity. Reusing water is a cause that he now champions professionally.

**Figure:**
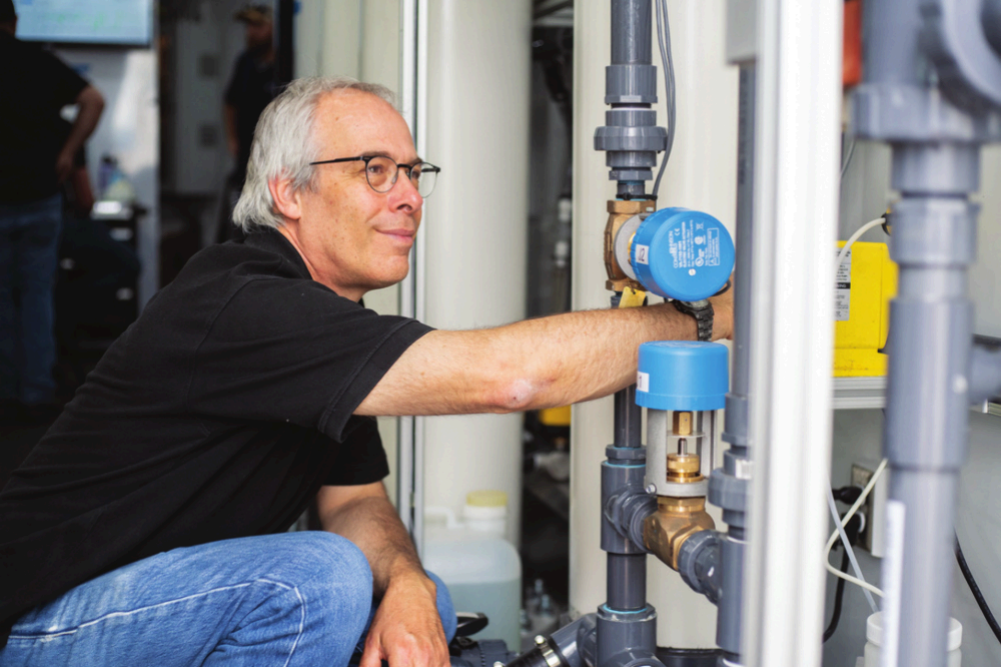
Environmental engineer Tzahi Cath works in a laboratory on wheels. Credit: Colorado School of Mines.

As part of his PhD project, Cath worked on ways to supply water to astronauts on a potential mission to Mars. One hundred forty million miles from home, the astronauts on Mars would have to use the same water they’d brought from Earth over and over again—including that from their toilets.

On Earth, such drastic measures might not seem necessary, but highly water-stressed areas such as Singapore, Southern California, and Texas are already reusing their wastewater to varying degrees. In 2019, the Colorado Springs Utilities approached Cath and said they wanted to show their customers what direct potable reuse might look like. Cath, by then an environmental engineer at the Colorado School of Mines, did not think twice. “I said immediately, ‘Yes, we are joining your group.’ ”

But instead of installing a test system at Colorado Springs Utilities’ wastewater treatment plant, Cath’s team built a mobile laboratory inside of a trailer. Cath has driven the lab, called the PureWater Colorado Direct Potable Reuse (DPR) Mobile Demonstration, around Colorado since 2021, showing wastewater treatment plants that they too can turn their effluent into potable water. XiaoZhi Lim spoke to Cath about the science behind the lab on wheels and the future of water reuse. This interview was edited for length and clarity.

## What inspired you to put the DPR lab on wheels?

We knew that if we were mobile, it’d be much easier to go from one place to another. The idea was to take it to the people, to places that might consider potable reuse. To see it in your city [and that the potable reuse system] works with your wastewater, I think people develop more trust in the process.

**Figure:**
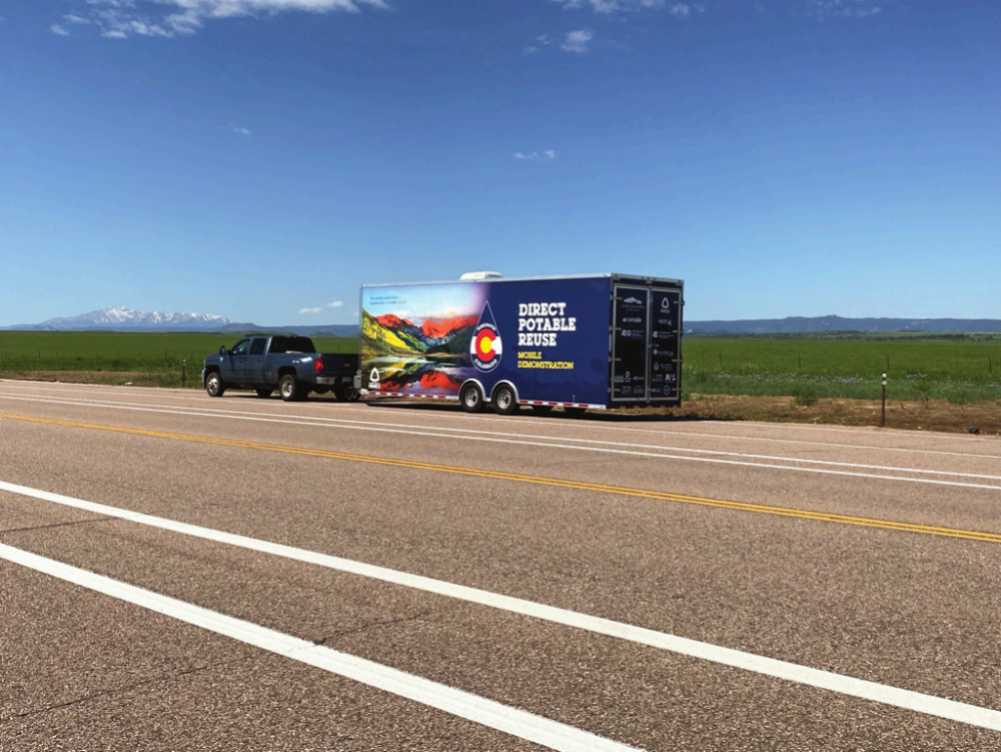
Colorado School of Mines researchers use their laboratory on wheels to show wastewater treatment plants how they can turn their effluent into potable water. Credit: Colorado School of Mines.

## Where have you taken the mobile lab so far?

We started in Colorado Springs, then we moved to the city of Aurora, then to Littleton and Englewood, and now we are in Denver. Since June 2021, now for more than 3 years, we are producing clean, drinkable water from effluent from municipal wastewater treatment plants.

## What are the key processes in the trailer?

[Our first process is] ozonation, mainly to break down some of the more recalcitrant organic compounds that are not degraded easily by microorganisms or other means. We add ozone in an approximately 1:1 ratio with the total organic carbon that comes into the trailer. After that, we put the water through biologically active filters—these are granular activated carbon columns that we allow microorganisms to grow on. These microorganisms are chewing on the organic matter that’s more available after the ozonation. After that, we put the water through ultrafiltration to remove [suspended solids and] any microorganisms that might have migrated from the biological process.

After that, we have a little split [in the water streams]. A reverse osmosis system takes a small side stream from the ultrafiltration step. The main stream goes through adsorption processes with granular activated carbon, ion-exchange resins, and an organoclay, with the latter two specific for removing PFAS [per- and polyfluoroalkyl substances].

Then we put the water [from both streams, separately] through UV [ultraviolet] irradiation with oxidation. We dose the water with peroxide before exposing it to UV light. That [exposure] generates hydroxyl radicals that break down other organics that managed to escape treatment. And in the last step, just before the water leaves, we add chlorine. In this trailer we can test side by side, on the same water at the same time, two approaches to potable reuse.

**Figure:**
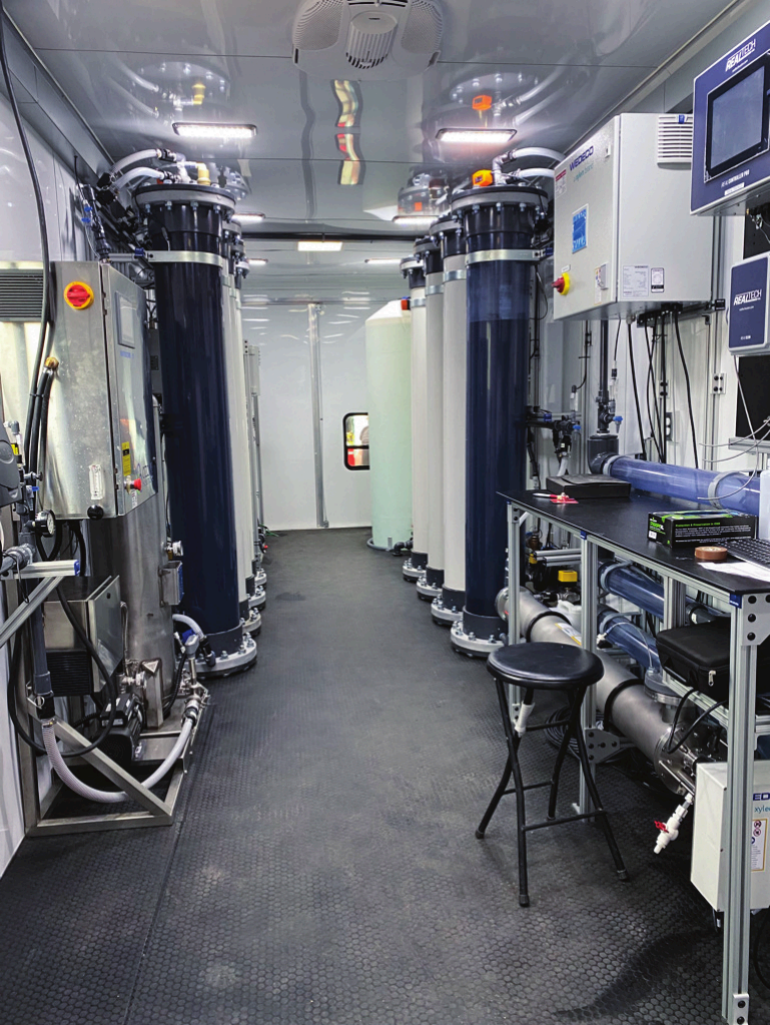
In the mobile lab wastewater moves through various treatment processes. Credit: Colorado School of Mines.

## Why is it important to test the two approaches to potable reuse?

The different states that are going to adopt potable reuse have different philosophies of how to do it. For example, California dictates that there should be reverse osmosis [RO]. One of the reasons is that they can send the concentrate [unwanted brine retained by the RO membrane] from RO to the ocean.

In Colorado, we don’t have an ocean, and it’s more difficult to deal with a brine. But if City X wants to implement DPR, we can come with the trailer and say: Here’s what’s left in the water after [treatment with RO], and here’s what’s left over after [treatment without RO]. Show it to your customers, and make a decision about which technologies you want to adopt for potable use. That’s the big thing of the ability to test side by side at the same time with the same water.

## How else do you plan to use the information from the trailer?

Another unique thing with what we are doing is related to Colorado’s Regulation 31. The regulation says that by 2037 the level of nitrogen in water discharged from wastewater treatment plants to the environment needs to be less than 2 mg/L. Today, the limit is 15 mg/L. Dissolved organic nitrogen is the main problem.

And so that’s what we are testing now—to see how well a treatment train, like the one we have, is doing in removing organic nitrogen. We have already demonstrated that we can get to less than 0.3 mg/L of organic nitrogen. So, the wastewater treatment plant will have to decide: Are we going to implement some processes just to [meet the new regulations], and then we’ll discharge to the river? Or let’s do potable reuse?

## Where are you headed to next, and what complications do you anticipate?

The next stop is going to be the Silicon Valley Clean Water reclamation plant, a small plant in Redwood City in California. One of the main challenges with moving to California is to cross two mountain ranges, the Sierra [Nevada] and the Rockies. But after we cross the mountains—and we won’t do it in the winter—we are going to focus on more difficult water to treat, that has more nitrogen, more phosphorus. We’ll have a lot of work there on comparing the two treatment trains [one using adsorption processes and one using RO]. In addition, we have a few other projects; one of the main ones is developing advanced control systems for optimal control of the treatment processes and early detection of failures.

## What is the biggest technical obstacle to potable reuse? Nontechnical?

I don’t think that there are technical issues. We know how to do it; the processes exist. We just need to make them more energy efficient, more chemically efficient, more resilient. The big thing is the economics part: How do you do it at the cost that people are willing and can buy this water?

And I think you need to educate the public that [potable reuse] is possible. We had a very good experience when we were in Colorado Springs in the first year. We had many public events; we allowed people to drink the water. We made some soda, we made some beer, and people realize that it tastes like regular water. Nobody dies, or nobody gets sick. It’s good water. I was the first one to drink the water from this trailer, and I’m still alive.

## XiaoZhi Lim is a freelance contributor to

Chemical & Engineering News, *the independent news outlet of the American Chemical Society.*

